# Supplementary material for: The influence of social networks in adoption of integrated health interventions: A qualitative study of fishermen in Malawi
Source: PLOS Glob Public Health. 2025 Jun 20;5(6):e0004581. doi: 10.1371/journal.pgph.0004581 (PMC12180632; doi:10.1371/journal.pgph.0004581)
Supplement: Attachment — Submitted filename: Response to Reviewers.docx [file pgph.0004581.s002.docx]

Helse Nord Tuberculosis Initiative

Kamuzu University of Health Sciences

Private Bag 360

Chichiri

Blantyre

Malawi

Mobile phone: +265888518464

Email: madalo.mukoka@gmail.com

8^th^ April 2025

Dear Editor,

It is with pleasure that we re-submit our research article titled “**The influence of social networks in adoption of integrated health interventions: a qualitative study of fishermen in Malawi**” following comments from reviewers.

All authors have seen and approved the revised manuscript. Please find below our responses:

Reviewer #1:

1. A great work highlighting the utility of social networks in influencing health decisions.

*Response: Thank you*

2. Your manuscript would benefit, especially in the discussion section, from a section that discusses current limitations in how social networks are used in health interventions and how your recommendations mitigate these limitations. A great way to start was when you stated that social network interventions should incorporate mechanisms to mitigate the spread of misinformation. You could for instance include recommendations on what these mechanisms could be. And then just as this recommendation directly addresses the limitation of misinformation in social networks, you can tailor your other recommendations to address other limitations.

*Response: Thank you for the comment and for providing guidance on how to respond. Firstly, we have added recommendations to the limitation on misinformation.* *(See lines 586-590)*

*‘****For example, pre-intervention education campaigns done in collaboration with trusted community leaders can be used to sensitize communities on anticipated barriers and address misinformation. Additionally, digital platforms can also be used to disseminate culturally tailored content and counter any negative rumors that can arise.’***

*Additionally, we had already included potential solutions to other limitations listed in the discussion*

*Second, it was challenging to qualitatively understand who is connected to whom and define information flow trajectories within the network.* ***Complementary quantitative methods are required to map the networks to visualize the social structure and analyze patterns of interaction, influence, and knowledge exchange.’ (see lines 599-604)***

*Lastly, the study was conducted in one district only, limiting the generalizability to other districts with different socio-cultural, economic, or healthcare contexts.* ***Future studies should consider recruiting from multiple contexts to enhance broader applicability.’ (See lines 604-607)***

Reviewer #2:

A well-conducted qualitative study examining the influence of social networks on Malawian fishermen's adoption of integrated health interventions is presented in this publication. For public health initiatives aimed at high-risk and mobile populations, the study is extremely significant. However, there is room for improvement in a few areas.

*Response: Thank you*

1. I am still unsure how social networks influence health behavior through mechanisms like social norms, social capital, and peer influence. Perhaps, a conceptual model or diagram illustrating how different levels of social networks impact health intervention adoption would be beneficial.

*Response: Thank you for your insightful comment. We acknowledge the importance of clearly illustrating how social networks influence health behavior through mechanisms such as social norms, social capital, and peer influence. We had included a conceptual diagram (see caption on line 123 and included in the submission package). We have updated the diagram to explicitly capture community level factors (e.g. social norms). This diagram illustrates how social interactions mediated by individual factors, wider community constructs (such as social norms, beliefs) and access to resources (social capital) generate social/peer influence which then impacts intervention adoption.*

*Individual factors determine who a person associates with and ultimately their social capital. The interactions enable information exchange and can generate peer influence which can determine whether an individual takes up an intervention or not.*

*Community level factors (e.g. norms, beliefs) shape the environment in which health behaviors occur. Community-level acceptance of health interventions and structural factors such as access to services impact the uptake of interventions.*

*Social influence, which is the central concept, aligns with the idea that individuals are shaped by their network connections. We hope that the diagram along with the narrative provided addresses the concerns. (see lines 117-121)*

2. A more clearer description of the coding framework used would help to refine the data analysis part.

*Response: Thank you for your feedback. We have now provided more detail to the description of the coding framework used in the analysis. We have provided better signposting to show the steps we followed from coding to theme generation. We believe this addition strengthens the clarity of the data analysis section. (see lines 185-209)*

***A codebook was generated using a hybrid approach- both deductive and inductive. Coding was performed by MM, OM and CC who received prior training using NVIVO by MK. The coding framework was informed by the conceptual framework, objectives and topic guides. Firstly, data familiarization was done, allowing for an initial understanding of the content and the identification of potential patterns. The initial coding was primarily deductive, guided by the conceptual framework and objectives before being imported into NVIVO (version 14) [33].***

***Parallel coding for each transcript was done and through this process, the priori codes initially incorporated in the coding framework were refined and expanded iteratively through an inductive process. This coding process involved reading and rereading each transcript, generating codes, team discussions, and comparison of emergent codes to ensure consistency. A total of 132 codes were generated in the process, however, not all of them were directly relevant to the focus of this write-up.***

***The next stage involved theme generation, which involved grouping and regrouping codes into broad thematic categories and then attaching theme labels to each distinct category. Through this process, the final codebook was structured hierarchically, with broad thematic categories at the top level and sub-themes nested beneath. The focus was on identifying themes that captured the essence of the participants' experiences and addressed the research objectives. The senior behavioral scientist (MK) played a critical role in this process by providing oversight and guidance throughout the coding and theme development stages. He facilitated coders’ training, discussions among coders to resolve discrepancies and provided methodological expertise in the transition from coding to theme generation. Finally, participants were provided with feedback on the findings.***

3. A table of the important key findings with themes, sub-themes and quotes would look more condensed and easy to read and understand.

*Response: Thank you for your suggestion. We appreciate the importance of presenting key findings in a clear and concise manner. To enhance readability, we have now included a table summarizing the key themes, sub-themes, and representative quotes (see lines 226-227).*

4. I would suggest compressing few quotes; some are too lengthy.

*Response: Thank you for this observation. We have shortened several quotes while ensuring that we do not change the meaning. (see lines 269, 317-320, 350-351, 356, 369-370, 444, 514-520, 530)*

5. Since the study was conducted in a single district, acknowledging this as geographic limitation would be useful.

*Response: Thank you for pointing this out. This has been acknowledged as a limitation and included in the discussion section. (See lines 604-607)*

***‘Lastly, the study was conducted in one district only, limiting the generalizability to other districts with different socio-cultural, economic, or healthcare contexts. Future studies should consider recruiting from multiple contexts to enhance broader applicability.’***

Reviewer #3:

Codebook Development: Please specify the total number of codes generated and provide a detailed description of the process used to create the codebook. Clarify as well the role of the senior researcher in this process. It would also be helpful to indicate which aspects of coding were influenced by a deductive versus an inductive approach.

Response: Thank you for this comment. We have revised the methods section to capture the different concerns

a) *Response: To develop the codebook, we took several steps as outlined in the analysis sub-section of the methods section. Firstly, we describe the deductive process of generating the initial codes (see line 185-191). Then, the inductive iterative process to expand on the priori codes (see lines 193-198). We also specified the number of codes generated through this process (see lines 197). Additionally, we explained how themes were generated and structured (see lines 199-204)*

***‘A codebook was generated using a hybrid approach- both deductive and inductive and was performed by MM, OM and CC who received prior data coding training using NVIVO by MK.’ (see lines 185-186)***

***‘A total of 132 codes were generated in the process, however, not all of them were directly relevant to the focus of this write-up*.” (See lines 197-198)**

b) *To clarify the role of the senior researcher in the coding process. We have included this statement*: (see lines 204-208)

***‘The senior behavioral scientist (MK) played a critical role in this process by providing oversight and guidance throughout the coding and theme development stages. He facilitated coders’ training, discussions among coders to resolve discrepancies and provided methodological expertise in the transition from coding to theme generation.’***

c) *In terms of deductive versus inductive coding, the initial coding framework was primarily deductive, as it was informed by the study’s conceptual framework, objectives, and topic guides. However, the iterative coding process that followed allowed for inductive expansion, as new codes emerged from the data and refining the initial framework. This hybrid approach ensured a comprehensive and flexible structure. We have explicitly mentioned the places where we used the deductive versus the inductive approach to provide more clarity.*

***“The initial coding was primarily deductive, guided by from the conceptual framework, objectives and transcripts, before being imported into NVIVO (version 14) [33].” (See lines 190-192)***

***“Parallel coding for each transcript was done and through this process, the priori codes initially incorporated in the coding framework were refined and expanded through an iterative inductive process.” (see lines 193-195)***

Sincerely

Madalo Mukoka
